# Desmoid tumors: clinical features and treatment options: a case report and a review of literature

**DOI:** 10.11604/pamj.2015.21.93.7037

**Published:** 2015-06-05

**Authors:** Amel Achour Jenayah, Hajer Bettaieb, Sarra Saoudi, Anissa Gharsa, Ezzeddine Sfar, Fethia Boudaya, Dalenda Chelli

**Affiliations:** 1 Department “A” of Gynecology and Obstetrics, Center of Maternity and Neonatology of Tunis, Tunisia

**Keywords:** Desmoïd tumor, surgery, radiotherapy, recurrence

## Abstract

Desmoid tumors are a rare group of locally aggressive, non malignant tumors of fibroblastic origin that can lead to significant morbidity due to local invasion and may even result in a fatal outcome when located around vital organs. Their clinical presentation, biological behavior and natural history can be quite varied and is incompletely understood at the present time. The optimal therapeutic approach depends on various factors, and a multidisciplinary approach is necessary to achieve local control with acceptable morbidity. Despite progress in the understanding of these tumors and the treatment options, local recurrence remains a major problem.

## Introduction

Desmoid tumors describe a rare monoclonal, fibroblastic proliferation characterized by a variable and often unpredictable clinical course. Although histologically benign, desmoids are locally invasive and associated with a high local recurrence rate, but lack metastatic potential. On the molecular level, desmoids are characterized by mutations in the catenin gene, CTNNB1, or the adenomatous polyposis coligene, APC. Many issues regarding the optimal treatment of patients with desmoids remain controversial; however, surgery is the therapeutic mainstay, except if mutilating and associated with considerable function loss. Postoperative radiotherapy reduces the local recurrence rate, in cases of involved surgical margins.

## Patient and observation

**Figure 1:**
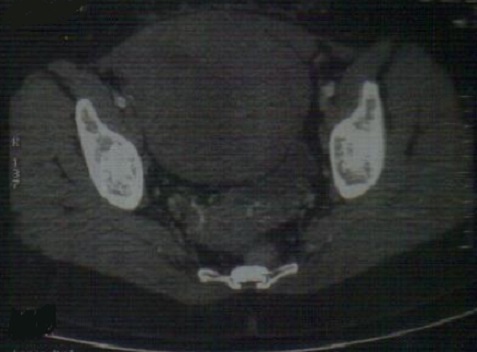
Abdomino-pelvic scan highlighting the mass (107x161x113 millimeters)

**Figure 2:**
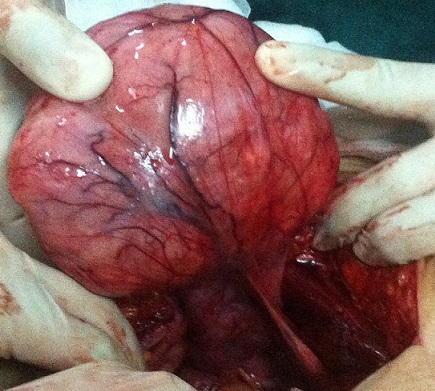
The operating observations: a fixed tumor to the peritoneum and to the fascia

A 27-year-old female (gravida 2, para 1) suffered with progressive abdomen distention associated with paroxystic pain since 2013. She doesn't consult till june 2014. No history of previous abdominal surgery, intestinal polyposis. Abdominal examination showed a firm nontender intra-abdominal mass, measuring around 10×14 centimeter size, with intrinsic mobility, mass mobile perpendicular to the mesenteric line, all borders were well made out. A clinical diagnosis of mass arising from mesentery was made. Abdomino-pelvic scan showed pelvic lesion tissue adherent to the posterior surface of the wall with the former and underside of the uterus and internal and external iliac vessels (107x161x113 millimiters) (Figure 1). Liver function test and renal function test were normal. Elective laparotomy was done showing a giant parietal mass with a huge infiltration of aponevrose which had necessitated a cure plate (Figure 2). Histopathology showed uniform band of spindle shaped cells arranged in fascicles admixed with blood vessels in a collagenous stroma. Cells were infiltrating skeletal muscle without necrosis and mitosis. Postoperative period was uneventful.

## Discussion

Desmoid tumors have an annual incidence of two to four cases per million, with an average age at diagnosis of 40 years and a slight female predominance [1]. The exact etiology of these tumors is unknown, but hormonal, genetic, and physical factors all play a role in their development and growth. Although the majority of desmoid tumors are idiopathic, associations with estrogens, pregnancy and trauma have been documented in literature [2]. They may occur sporadically or in association with familial adenomatous polyposis (FAP). Approximately 7,5% of cases are associated with familial adenomatous polyposis (FAP) in the general population, while studies from a tertiary institution may see this rate being as high as 15%. On the other hand 12-15% of patients with FAP develop desmoid tumors. Desmoid tumors most commonly involve the extra abdominal locations in the general population whereas patients with FAP mostly present with intraabdominal disease. Mesenteric desmoid tumors are known to be the second leading cause of death in FAP patients. There is inconsistency in the literature regarding the risk factors for desmoid tumors in FAP [3]. In general a positive family history, an APC mutation 3’ to codon 1399, previous abdominal surgery and the female sex have been implicated as risk factors in FAP [3]. Desmoid tumors may affect all sites, including the extremities, trunk, and abdomen [4]. For example, only 5% of sporadic desmoid tumors are intra-abdominal, but 80% of patients with familial adenomatous polyposis (FAP)-associated desmoid tumors develop intra-abdominal disease. The incidence is 3% of soft tissue sarcomas and about 0,03% of all malignancies [1]. Desmoids are therefore a distinct rare tumor entity and are seen in about three to four cases per 1 million of the U.S. population. Desmoids occur between the age of 15 and 60 years, but particularly during early adolescence, and with a peak age of about 30 years. Two different types of desmoid tumors are described: besides the sporadic desmoid tumor manifestation, there is a special relationship between desmoids and FAP (Gardner syndrome). An incidence of 3,5%-32% has been reported in these patients [2]. The usual presentation is that of a slow growing mass without associated pain or discomfort. Depending on location the tumor may present with symptoms such as neurological deficit, joint stiffness or abdominal complaints. In our case, the patient consulted because of abdominal pain and inspite of the important size of the mass, the abdominal pain was not huge and patient had delayed for consulting. While desmoid tumors are known to spontaneously regress in a few cases, many continue to progress and need to be treated [5]. There is also evidence for varying periods of growth in the life of a lesion, including a stable phase. Desmoid tumors are histologically benign tumors that do not metastasize. However they can be locally aggressive and hence the main goal of desmoid treatment is local control. Extra abdominal tumors are not commonly associated with mortality however morbidity and disfigurement may be related to treatment or tumor progression. The literature lacks level I evidence in the form of randomized controlled trials to compare the relative efficacy various treatment modalities. A multitude of treatment options are available and choosing the appropriate method for achieving local control depends on the functional and cosmetic outcomes of each method and the associated complications [6, 7]. The natural history of desmoids tumors continues to be an enigma. Desmoids have been reported to remain stable for prolonged periods of time or even regress spontaneously in some reports. The surgical challenges and morbidity associated with the treatment of desmoid tumors have forced a change in global trends toward adopting a proconservative approach. A “wait and watch” approach has been promoted by several recent studies [8]. An improved understanding of the molecular biology of these tumors may also help in identifying patients at risk for recurrence [9]. Adjuvant treatment modalities may be utilized early in these high risk cases.

The infiltrative and recurrent nature of desmoid tumors can render surgical resection challenging if acceptable function and cosmesis is to be maintained. Aggressive attempts at resection have the potential to make the treatment worse than the disease. Treatment in each case has to be individualized with multidisciplinary participation [9]. Acceptable levels of function and cosmesis will vary from patient to patient depending on location, occupation, handedness, prior level of functioning and morbidity caused by the disease itself. It is a determination that is finally made by the patient in concert with the health care providers. The plastic surgery team, occupational therapists, physical medicine and rehabilitation specialists and members from the amputee service can provide valuable input to help with this decision. In order to avoid the morbidity of surgery or radiotherapy, a period of watchful waiting may be the most appropriate management in selected patients. The current trend is strongly in favor of treating asymptomatic desmoids with observation, only reserving treatment for those tumors that may pose danger to vital structures or show continued growth [10]. Systemic therapy is an option in unresectable or recurrent disease. Available options include hormonal therapies, nonsteroidal anti-inflammatory drugs (NSAIDs), interferon, and chemotherapy. The use of hormonal therapy for the treatment of these tumors is based on the association of these tumors with pregnancy or contraceptives pills and reports of regression after menopause or oophorectomy. Success rates of around 50% have been obtained with hormonal treatments and other agents such as NSAIDs, Vitamin C, and warfarin. The most common regimen uses high dose tamoxifen at 120 mg per day along with sulindac. Response and control rates have been reported to be over 50% [11]. Some patients cannot tolerate this treatment or fail to respond. Such patients with symptomatic, progressive disease who can tolerate chemotherapy can be managed with either low-dose or standard antisarcoma chemotherapy. The use of low-dose methotrexate and vinorelbine for up to one year has been shown to control desmoids with manageable side effects. More aggressive standard antisarcoma systemic therapy with doxorubicin- or ifosfamide-based chemotherapy is also effective in desmoids. Although it is unclear what the optimal regimen is, patients appear to have quicker responses to the standard antisarcoma therapy especially with regimens containing doxorubicin [11]. The use of chemotherapy has to be weighed against the potential for morbidity. The use of tyrosine kinase imatinib has shown some moderate control of desmoids [11]. The recent positive reports, of sorafenib in desmoid will need to be confirmed by other groups [10]. Tamoxifen an antagonist of the estrogen receptor is currently being studied for effectiveness in hormone receptor positive breast desmoid tumors. Newer research suggests that deregulation of the mammalian target of rapamycin (mTOR) cell proliferation/survival pathway may play an important role in desmoid tumor biology especially when the APC/β-catenin pathway is disrupted. Several randomized controlled trials are currently recruiting patients to study the role of these newer systemic treatment modalities for desmoid tumors [10]. Retrospective studies indicate that radiation therapy may improve the local control of desmoid tumors, in both the adjuvant and the primary setting [10]. A review of literature concluded that the rates of local control with either surgery with radiotherapy or radiotherapy alone are significantly better than surgery alone regardless of the margins achieved at surgery [3]. The relative superiority of radiotherapy alone or combined with surgical management is amplified in cases with positive margins. This underscores the importance of combined modality management in tumors with positive margins and that of radiotherapy alone in tumors which are unresectable [10]. This does come at the cost of short term and long term radiotherapy related complications which are observed in 17% of patients especially with radiation doses higher than 56 Gray [10]. The best results are associated with high dose radiation and it may take up to two years for the tumor to regress [10]. The rate of local recurrence associated with radiotherapy is significantly increased when radiotherapy is used as the sole modality in doses less than 50 Gray. No differences in the rate of local recurrence are observed with high or low dose radiotherapy when combined modality management is utilized [12]. The most common complications observed with the use of radiotherapy are fibrosis, paresthesias, edema, fractures and local skin irritation [12]. Surgical resection is the primary treatment modality for desmoid tumors when functionally and cosmetically acceptable with reported local control rates of 75-80% [12]. It is also recommended when the tumor is close to vital structures and progression would be associated with high morbidity or mortality [13]. Despite the reported high local control rates with surgery alone, local recurrence rates have varied from 24-77% between series [13]. Although margins are thought to influence local control in soft tissue sarcoma, the literature on desmoid tumors presents conflicting evidence. While some studies have stressed on the futility of aggressive surgery to obtain negative margins, other studies have shown that margins do influence local recurrence rates [14]. A recent comparative analysis of the available literature concluded that wide surgical margins significantly influence recurrence in surgically treated desmoid tumors [14]. Contrarian opinions regarding this issue; sometimes from the same institutions; underlines the importance of issues such as selection bias and study design in retrospective studies [15]. No randomized controlled trials are available as yet to guide treatment. Surgery is also recommended in recurrent cases whenever feasible since local control rates are similar to primary surgical excision. In the case of patients with unresectable extremity desmoids tumors function preserving procedures should be the goal. In case of patients who have failed systemic therapy and/or radiation and whose only option is amputation it may be possible to safely follow them unless the limb is painful, functionless or infected [15].

## Conclusion

Desmoid tumors are locally aggressive fibrous tissue tumors with a tendency for local recurrence despite adequate surgical resection. Asymptomatic or non-progressive tumors may be carefully observed. Surgical resection is favored when functionally and cosmetically acceptable however combined modality management is useful in recurrent or unresectable tumors. Radiation may be indicated after margin positive resection or if unresectable with impending functional problems. Systemic therapies should also be considered in cases where the tumor is unresectable, especially in cases where radiation toxicity may be also unacceptable.
